# Specific Inhibitory Effect of κ-Carrageenan Polysaccharide on Swine Pandemic 2009 H1N1 Influenza Virus

**DOI:** 10.1371/journal.pone.0126577

**Published:** 2015-05-13

**Authors:** Qiang Shao, Qiang Guo, Wen ping Xu, Zandong Li, Tong tong Zhao

**Affiliations:** State Key Laboratory for Agrobiotechnology, College of Biological Sciences, China Agricultural University, No. 2 Yuan Ming Yuan West Road, Beijing, 100193, China; US Food and Drug Administration, UNITED STATES

## Abstract

The 2009 influenza A H1N1 pandemic placed unprecedented demands on antiviral drug resources and the vaccine industry. Carrageenan, an extractive of red algae, has been proven to inhibit infection and multiplication of various enveloped viruses. The aim of this study was to examine the ability of κ-carrageenan to inhibit swine pandemic 2009 H1N1 influenza virus to gain an understanding of antiviral ability of κ-carrageenan. It was here demonstrated that κ-carrageenan had no cytotoxicity at concentrations below 1000 μg/ml. Hemagglutination, 50% tissue culture infectious dose (TCID_50_) and cytopathic effect (CPE) inhibition assays showed that κ-carrageenan inhibited A/Swine/Shandong/731/2009 H1N1 (SW731) and A/California/04/2009 H1N1 (CA04) replication in a dose-dependent fashion. Mechanism studies show that the inhibition of SW731 multiplication and mRNA expression was maximized when κ-carrageenan was added before or during adsorption. The result of Hemagglutination inhibition assay indicate that κ-carrageenan specifically targeted HA of SW731 and CA04, both of which are pandemic H1N/2009 viruses, without effect on A/Pureto Rico/8/34 H1N1 (PR8), A/WSN/1933 H1N1 (WSN), A/Swine/Beijing/26/2008 H1N1 (SW26), A/Chicken/Shandong/LY/2008 H9N2 (LY08), and A/Chicken/Shandong/ZB/2007 H9N2 (ZB07) viruses. Immunofluorescence assay and Western blot showed that κ-carrageenan also inhibited SW731 protein expression after its internalization into cells. These results suggest that κ-carrageenan can significantly inhibit SW731 replication by interfering with a few replication steps in the SW731 life cycles, including adsorption, transcription, and viral protein expression, especially interactions between HA and cells. In this way, κ-carrageenan might be a suitable alternative approach to therapy meant to address anti-IAV, which contains an HA homologous to that of SW731.

## Introduction

In late April 2009, the emergence of the pandemic H1N1/2009 virus in North America caused a pandemic, which attracted a great deal of attention from all over the world [1,2]. Some 284,500 human deaths were caused by the virus in the first 12 months of circulation [3]. Swine, the intermediate host and reservoir of influenza virus, play a critical role in the transmission, prevalence, and recombination of H1N1/2009 influenza viruses [4]. In China, natural isolates of H1N1/2009 influenza viruses, which were more virulent than the early representative isolate (CA04) were identified in swine [5]. The naturally occurring mutations in PA gene are the critical cause of the increasing virulence of H1N1/2009 viruses [5]. Although various vaccines and antiviral drugs are available to protect humans and cure them of influenza virus infection, other antiviral agents capable of addressing problems caused by rapidly emerging H1N1 isolates, which have high rate of resistance mutations, are urgently needed.

Carrageenan, a high-molecular-weight sulfated polysaccharides derived from red algae, show different inhibitory effects on different viruses, including dengue virus [6], herpes simplex virus (HSV) [7], human rhinovirus (HRV) [8], human papillomavirus (HPV) [9], human immunodeficiency virus (HIV) [10,11], hepatitis A virus (HAV) [12], enterovirus [13], and IAV [14,15]. Three forms of carrageenan have been identified: kappa (κ), iota (ι), and lambda (λ). These differ in degree of sulfation, solubility, and gelling properties [16]. Carrageenan was found to inhibit the replication of HPV [9], rhinovirus [8], and dengue virus [6] by interfering with viral adsorption and internalization into host cells. However, C.A. Pujol et al reported that carrageenan could also inhibit some steps of HSV life cycle in host cells instead of adsorption and internalization [17]. Carrageenan has also shown virucidal activity on some enveloped viruses such as HSV-2 [18]. Recently, Wang et al. demonstrated that 2 kDa κ-carrageenan oligosaccharide can inhibit influenza H1N1 viral multiplication by inactivating viral particles, repressing transcription and protein expression without affecting adsorption [14,15]. However, Leibbrandt et al. reported that ι-carrageenan polysaccharide could interfere with the attachment between H1N1 virus and Madin-Daby canine kidney cells (MDCK) [15]. The inhibitory mechanism of carrageenan on viral replication seems to be dependent on the degree of sulfation of polysaccharide, molecular weight, the serotype of the virus, and the host cells.

The purpose of the present work was to develop new methods of controlling IAV. It is here hypothesized that κ-carrageenan polysaccharide may provide effective protection against SW731. In the present study, the molecular mechanisms by which κ-carrageenan inhibits SW731 infection were investigated. The current results indicated that κ-carrageenan could inhibit SW731 replication in a dose-dependent fashion and that it did so effectively. κ-carrageenan did not interfere with virus receptor on the host cell surface, but it did interfere with adsorption by specific targeting the HA of SW731. κ-carrageenan also inhibited SW731 mRNA and protein expression after internalization into cells.

## Materials and Methods

### Compounds and Reagents

κ-carrageenan polysaccharide was purchased from 3 Source of Rong Yuan F.F.I.co., Ltd. The dry power was dissolved in phosphate buffer (PBS) to a concentration of 10,000 μg/ml as stock solution. Then the solution was sterile filtered through a 0.22 μm filter (Millipore, U.S.) and stored at 4°C until use. Ribavirin (Sigma-Aldrich) served as a positive control. Anti-NP protein mouse monoclonal antibody was from Abcam (U.S.). Anti-GAPDH monoclonal antibody and HRP-labeled goat anti-mouse secondary antibody were purchased from Beijing Co Win Biotech (China). FITC-labeled goat anti-mouse secondary antibody was obtained from Millipore (U.S.).

### Cell culture and virus infection

MDCK cells were maintained in Dulbecco’s modified Eagle’s medium (DMEM) supplemented with 10% FBS (Gibco, U.S.) and antibiotics. SW731, PR8, WSN, ZB07, SW26, CA04, and LY08 viruses were amplified in MDCK cells. The titers were determined to TCID_50_ using IFA. For viral infection, viral propagation solutions were diluted in DMEM and added to cells at the indicated multiplicity of infection (MOI). After adsorption for 60 min at 4°C, viral inoculum was removed and cells were maintained in infecting media (DMEM) containing 2 μg/ml tosylsulfonyl phenylalanyl chloromethyl ketone (TPCK)-trypsin (Worthington Biochemical, Lakewood, NJ, U.S.), at 37°C in 5% CO_2_.

### Cytotoxicity assays

The cytotoxicity of κ-carrageenan was measured using the MTT assay (3-[4,5-dimethylthiazol-2-yl]-2,5-diphenyl tetrazolium bromide; Sigma-Aldrich, U.S.). MDCK cells were seed at a density of 1×10^4^ cells per well of a 96-well plate in 100 μl of complete growth medium overnight. MDCK cells were exposed to different concentrations of compounds in triplicate at 37°C in 5% CO_2_. Then, 48 h later, 10 μl of PBS containing MTT (final concentration: 0.5 mg/ml) was added to each well. After 4 h of incubation at 37°C, the supernatant was removed and 200 μL of DMSO was added to each well to solubilize the formazan crystals. After vigorous shaking, absorbance values were measured in a microplate reader (Bio-Rad, U.S.) at 570 nm.

### Hemagglutination (HA) assays

Then 25 μl SW731, CA04, PR8, WSN, and ZB07 viruses were serially diluted in the blood clot count plate. Then each sample was mixed with 25 μl of 1% chicken red blood cells (RBC) and incubated at room temperature. Then, 30 min later, the HA titers were determined.

### Viral titer measurement

The viral titer was measured with the TCID_50_. Briefly, the viral solution was serially diluted 10-fold in DMEM. A 100 μl aliquot of each diluted sample was added to the wells of 96-well plates, containing monolayer MDCK cells. Cells were cultured for 36–48 h at 37°C in 5% CO_2_. NP protein positive wells in IFA were considered positive. The titer was calculated using a previously described method [19]. All experiments with virus were performed under biosafety level 2 (BSL-2) conditions with investigators wearing appropriate protective equipment in compliance with general biosafety standards for microbiological and biomedical laboratories of Ministry of Health of the People’s Republic of China (WS 233–2002).

### CPE inhibition assay

Then 2.5 × 10^5^ MDCK cells were seeded in 96-well plates and maintained in DMEM plus 10% FBS overnight. After infection with SW731 at an MOI of 1, the cells were treated with κ-carrageenan at indicated concentrations and incubated at 37°C in 5% CO_2_. Then, 48 h later, the CPE was determined as previously described. Briefly, MDCK cells were fixed with 4% formaldehyde for 20 min at room temperature. After removal of the formaldehyde, the cells were stained with 0.1% crystal violet for 30 min and then washed and dried. Then the plates were solubilized with methanol and the intensity of crystal violet staining for each well measured at 570 nm. The concentration required for a test compound to reduce the CPE of the virus by 50% (IC50) was determined.

### Quantitative real-time PCR (qRT-PCR)

Total RNA was extracted from SW731 infected MDCK or RAW cells using a TRIgene (GeneStar, China), and treated with DNase I (Promega, U.S.) to remove the contaminating DNAs. Then 1 μg of total RNA was reverse-transcribed into cDNA using a GoScript reverse transcription system (Promega, U.S.) in a 20 μl reaction mixture. The cDNA was analyzed by qRT-PCR using SYBR green Master I (Roche, U.S.). QRT-PCR was performed with the following specific primers: NS1, 5′-CAACACCCTTGGCCTCGATA-3′ and 5′- AGTGTCTCGCTGGATTCCTCTT-3′ (GenBank accession No. J02150); NP, 5′-GCTGCGGTGAAAGGAGTTG-3′ and 5′-TTCACCCCTCCAGAAATTTCG -3′ (GenBank accession No. EF190975); canine β-actin 5′-GGCATCCTGACCCTGAAGTA-3′ and 5′-GGGGTGTTGAAAGTCTCGAA-3′ (GenBank accession No. XM536230); Real-time quantitative PCR was performed under the following cycling conditions: 95°C for 10 min for activation followed by 40 cycles at 95°C for 15 s and 60°C for 1 min. This was followed by one cycle of 95°C for 15 s, 60°C for 15 s, and 95°C for 15 s. The final step was to obtain a melt curve for the PCR products to determine the specificity of amplification. All controls and infected samples were carried out in triplicate on the same plate. Relative mRNA abundances were calculated using the 2^-ΔΔCt^ method with β-actin as a reference and plotted as fold change relative to mock-control samples [20].

### Indirect immunofluorescence assay

MDCK cells were washed with PBS and fixed with 4% paraformaldehyde for 10 min. Then cells were permeabilized with 0.5% (v/v) Triton X-100 in PBS for 10 min before incubated with 3% BSA/PBS for 1 h at 37°C. Cells were washed and incubated with anti-NP antibody for overnight at 4°C. After three rounds of washing, the cells were incubated with FITC-labeled secondary antibody for 1 h at 37°C. Cells were examined with an inverted fluorescence microscope (Olympus, Japan).

### Western blot analysis

MDCK cells were lysed in RIPA containing 20 mM Tris base-HCl (pH 7.5), 150 mM NaCl, 1.0 mM EDTA (pH 8.0), 1.0 M EGTA (pH 8.0), 0.1% SDS, 0.5% DOC (Sigma, U.S.), 1% NP-40 (Sigma, U.S.), protease inhibitor cocktail (Roche, U.S.), and phosphatase inhibitor cocktail (Roche, U.S.). Proteins were separated by SDS-PAGE under reducing conditions and analyzed by Western blotting using anti-NP, anti-GAPDH antibodies and HRP-labeled secondary antibodies [21].

### Hemagglutination Inhibition assay

Then 25 μl of serially diluted SW731, SW26, CA04, WSN, LY08, and ZB07 virus were incubated with the same volume of PBS (Mock) or serially diluted κ-carrageenan for 20 min. Then each sample was mixed with 50 μl of 1% RBC at room temperature. After 30 min, the concentrations of κ-carrageenan inhibiting HA activity were measured.

### NA enzyme assay

NA activity was analyzed using a method which has been previously described [22]. Briefly, SW731 virus stock was diluted to 10^5^ pfu/50 μl with 33 mM MES (2-[N-morpholino]ethanesulfonic acid) buffer pH 6.5 containing 4 mM CaCl2 in the presence or absence of κ-carrageenan. NA activity assay was carried out at 37°C using methylumbelliferyl-N-acetylneuraminic acid (MUNANA) as substrate and luorescence was quantified on a fluorimeter.

### Statistics

All data analyses were performed using *SPSS 16*.*0*. Independent sample *t*-testing was used to detect significant differences between compound-treated groups and control groups. *P* values **<** 0.05 were considered significant. Data are presented as mean ± SEM.

## Results

### κ-carrageenan treatment does not show harmful effects on cell metabolic activity

**Fig 1:**
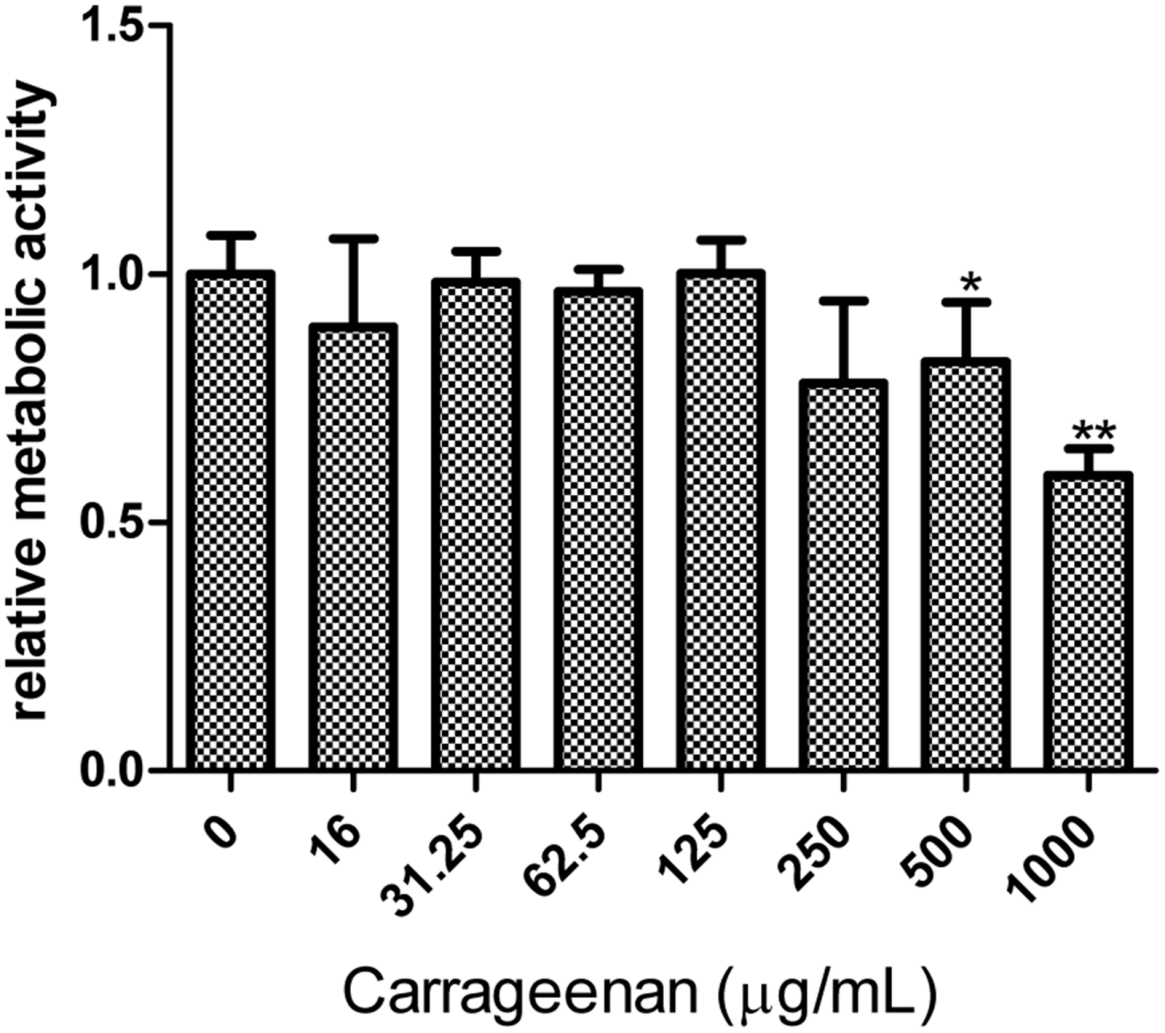
Cytotoxic effect of κ-carrageenan on MDCK cells. MDCK cells were treated with the indicated concentrations of κ-carrageenan. After 48 h of incubation, metabolic activity was measured via MTT assay. Results were analyzed with the independent sample t test (n = 3). Values are means ± SEM (n = 3). Significance: *P < 0.05 vs. nondrug-treated control group; **P < 0.005 vs. nondrug-treated control group. Results are representative of two independent experiments.

Because the use of drugs targeting cellular factors raises concerns about side effects on the host cells, antiviral-acting concentrations of κ-carrageenan were assessed for cytotoxicity. MTT assays were performed after 48 h of κ-carrageenan treatment at the indicated concentrations to investigate the impact of κ-carrageenan treatment on the metabolism of MDCK cells. There was no observable difference in metabolic activity of MDCK cells treated with concentration of 62.5–250 μg/ml κ-carrageenan and untreated cells (CC_50_ > 1000 μg/ml) (Fig 1). But the concentration of 1000 μg/ml κ-carrageenan treatment led to a reduced relative metabolic activity 62% (Fig 1).

### κ-carrageenan specifically inhibits SW731 multiplication in MDCK cells

**Fig 2:**
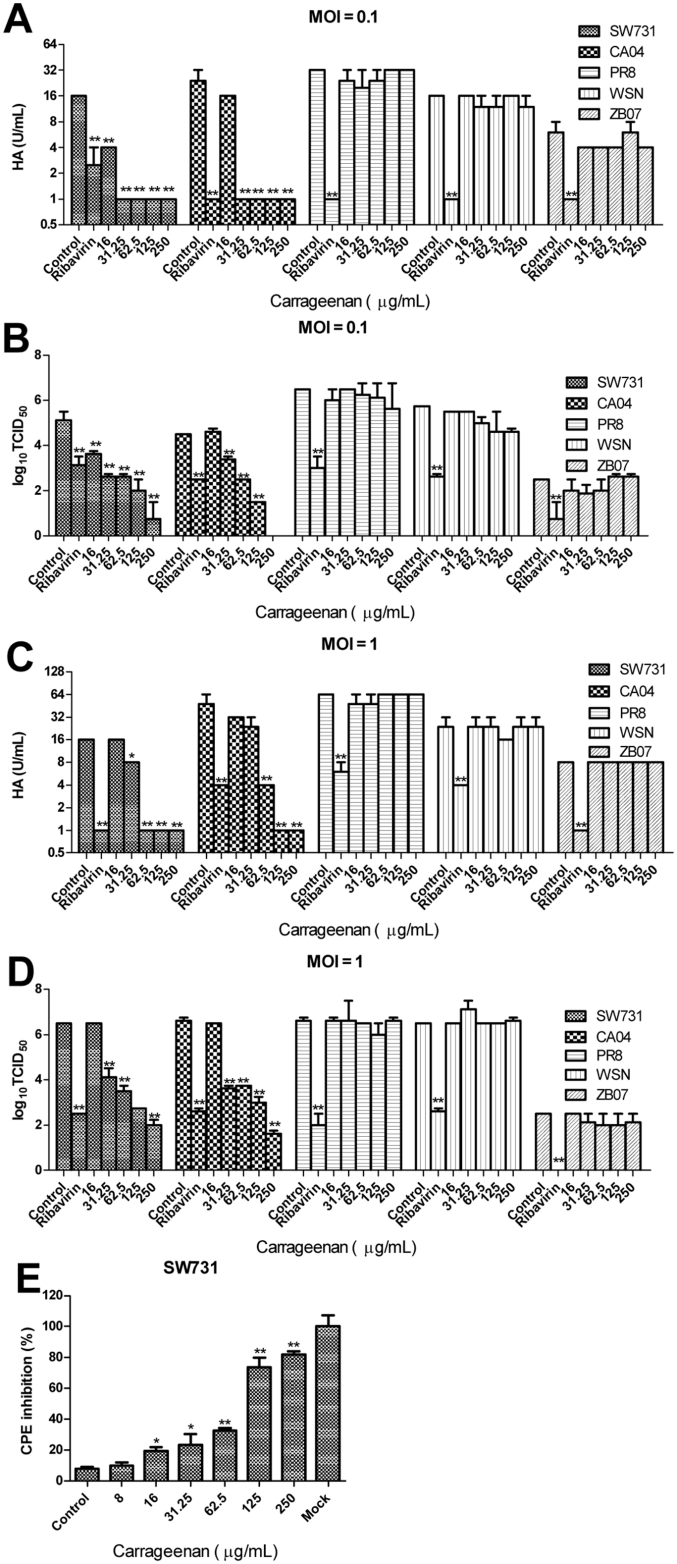
Anti-SW731 effect of κ-carrageenan on MDCK cells. (A, B) MDCK cells were incubated with SW731, CA04, PR8, WSN, and ZB07 at 4°C (MOI = 0.1) and then treated with κ-carrageenan or Ribavirin at the indicated concentration after removal of the virus inoculum. After 24 h, the viral titers were determined by HA and TCID50 assays. (C, D) MDCK cells were incubated with SW731, CA04, PR8, WSN, and ZB07 at 4°C (MOI = 1) and then treated with κ-carrageenan or Ribavirin at the indicated concentration after removal of the virus inoculum. After 24 h, the viral titers were determined by HA and TCID_50_ assays. (E) MDCK cells were infected with SW731 at an MOI of 1 and then treated with κ-carrageenan at the indicated concentration after removal of the viral inoculum. After 48 h, CPE inhibition was determined by CPE assay. Results were analyzed with the independent sample t test (n = 3). Values are means ± SEM. Significance: *P < 0.05 vs. nondrug treated control group; **P < 0.005 vs. nondrug treated control group. Results are representative of two independent experiments.

To investigate the in vitro anti-IAV activity of κ-carrageenan, MDCK cells were first infected with different strains of IAV (SW731, CA04, PR8, WSN, and ZB07) at an MOI of 0.1 or 1 and then treated with κ-carrageenan at the indicated concentrations. After 24 h, virus titers of the supernate were determined using TCID_50_ and HA assays. Fig 2A–2D show that the κ-carrageenan could markedly decrease the virus titer of SW731 and CA04 in a dose-dependent manner, while, PR8, WSN and ZB07 are not sensitive to κ-carrageenan. Only 250 μg/ml κ-carrageenan showed even a slight inhibitory effect on viral titer of WSN with a lower initial MOI of 0.1. CPE inhibition was performed to testify the protective effect of κ-carrageenan on MDCK from SW731. Consistent with the viral titer assay, it was here observed that the cell viability was significantly promoted by κ-carrageenan in a dose-dependent fashion (Fig 2E) with an EC_50_ value of 89.57±2.1 μg/ml. In this way, κ-carrageenan specifically suppressed the replication of SW731 instead of the classical H1N1 strain PR8, WSN and avian influenza virus ZB07 in MDCK cells.

### κ-carrageenan interfered with extracellular and intracellular steps in SW731 replication

**Fig 3:**
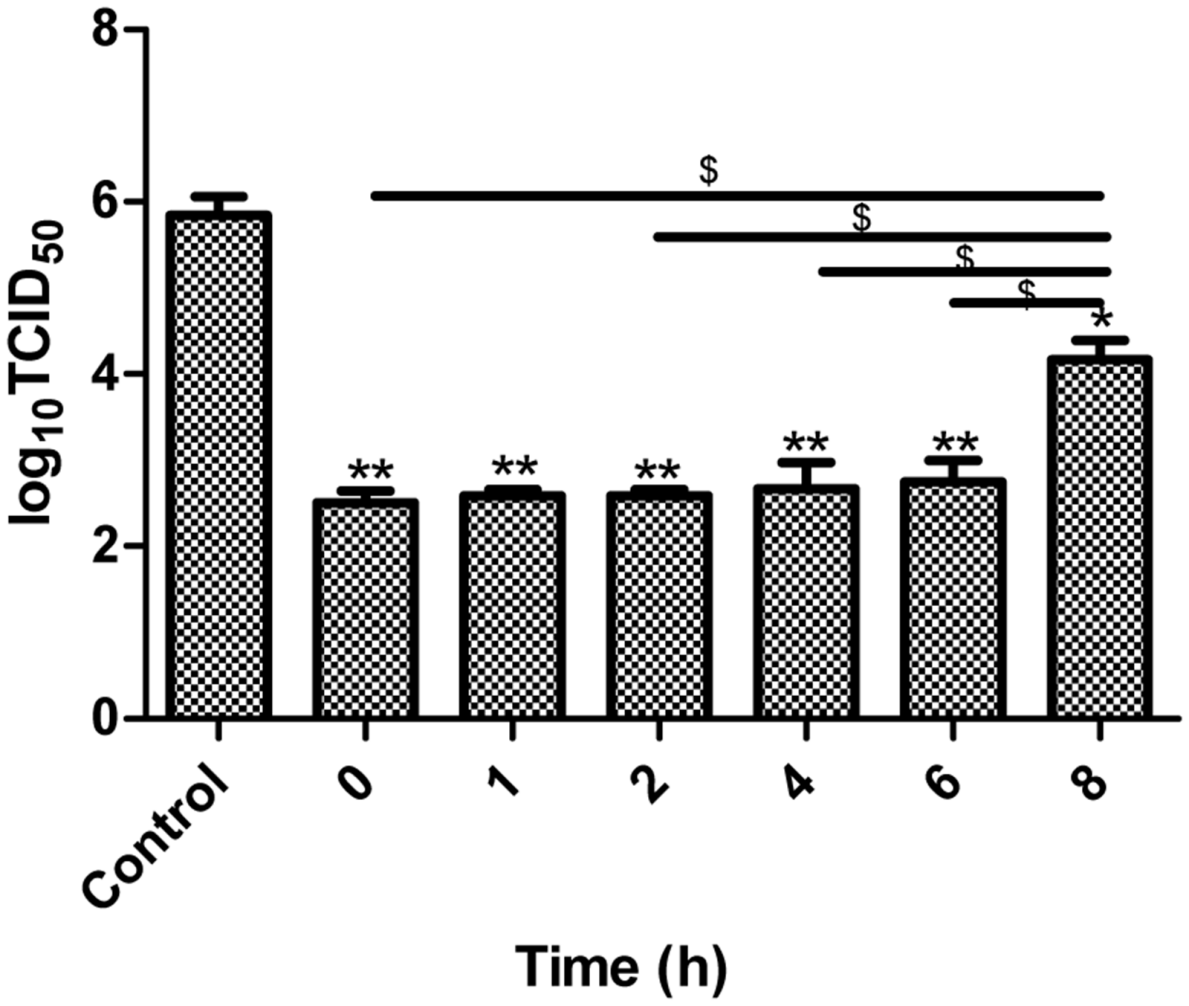
Time of addition of κ-carrageenan on SW731 replication. MDCK cells were infected with SW731 at an MOI of 3. κ-carrageenan was added to the cells at 0 h (adsorption), 1 h, 2 h, 4 h, 6 h, and 8 h. After 24 h, viral titers were determined using TCID_50_ assays. Results were analyzed with the independent sample t-test (n = 3). Values are means ± SEM. Significance: *P < 0.05 vs. nondrug treated control group; **P < 0.005 vs. nondrug treated control group. ^*$*^ *P <* 0.05 vs. 8 h group. Results are representative of two independent experiments.

To determine which steps of SW731 replication are involved in inhibitory actions of κ-carrageenan, κ-carrageenan was added to MDCK cells for an 8 h period of overall viral adsorption (0 h), internalization (1 h), early replication (2–6 h), and release (8 h). Then, 24 h p.i., the inhibitory effect was assessed with TCID_50_ assays. As shown in Fig 3, viral titers were markedly lower when κ-carrageenan were added at 0, 1, 2, 4, and 6 h.p.i., and κ-carrageenan could not effectively inhibit SW731 multiplication when κ-carrageenan were added at 8 h.p.i. No difference was observed among the viral titers when κ-carrageenan was added at 0, 1, 2, 4, and 6 h.p.i. These data indicate that a few of the extracellular and intracellular steps of SW731 replication were targets of κ-carrageenan.

### κ-carrageenan directly inhibited SW731 adsorption and internalization

**Fig 4:**
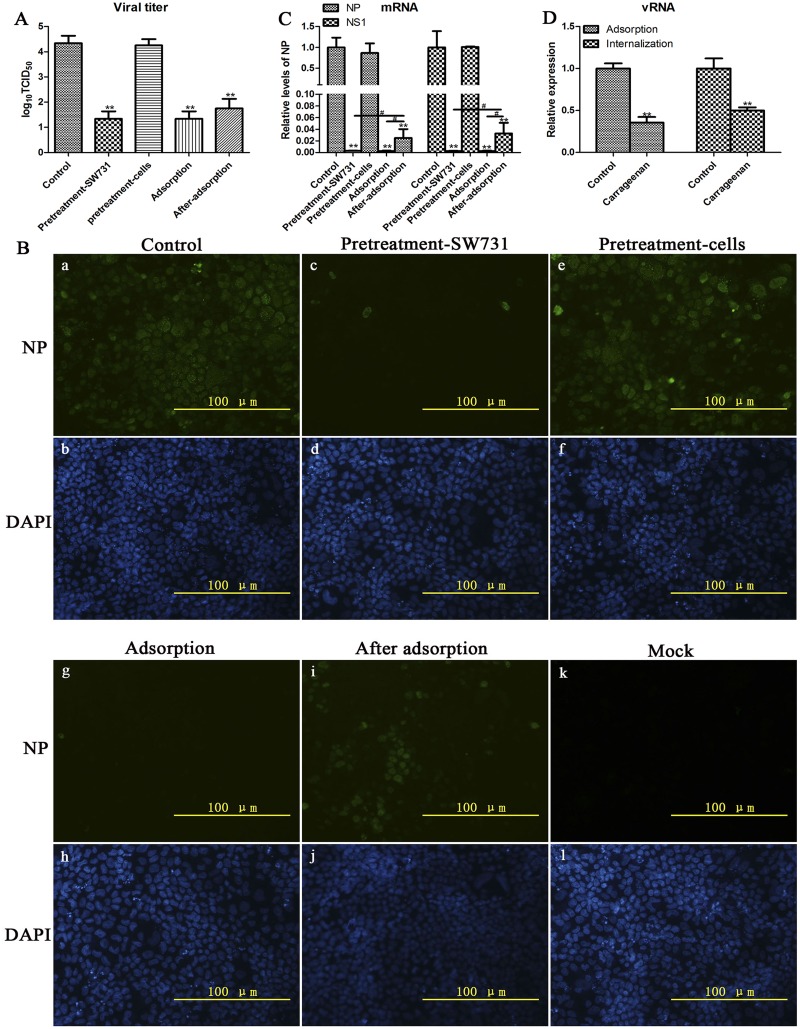
κ-carrageenan-induced inhibition of viral adsorption and internalization. (A–C) MDCK cells were infected with SW731 (MOI = 3) under 4 different treatment conditions. (i) Pretreatment-SW731: SW731 was mixed with 250 μg/ml of κ-carrageenan at 4°C for 1 h before adsorption. Then the virus/compound mixture was added to cells for 1 h at 4°C, and the media were removed and washed with PBS for three times to remove the compound. The cells were maintained in infective media at 37°C for 24h; (ii) Pretreatment-cells: MDCK cells were treated with 250 μg/ml of κ-carrageenan at 37°C for 1 h before infection. After removing the compound, cells were washed with PBS and incubated with virus for 1 h at 4°C. Then cells were maintained in infective media at 37°C for 24h; (iii) Adsorption: cells were incubated with virus/carrageenan mixture for 1 h of adsorption at 4°C. Then cells were washed and overlaid with infective media at 37°C for 24 h; (iiii) After-adsorption: cells were incubated with virus for 1 h at 4°C. After removal of unabsorbed virus, cells were overlaid with infective media containing 250 μg/ml of κ-carrageenan for 1h at 37°C. Then cells were washed with PBS and maintained in compound free infective media for 24 h. Viral titers were determined in a series of TCID50 assays. The mRNA levels and NP-positive cells were detected using qRT-PCR and immunofluorescence staining, respectively. (D) MDCK cells were infected with SW731 (MOI = 3) by 2 different treatment conditions. (i) Adsorption: MDCK cells were incubated with SW731 in the presence or absence (control) of κ-carrageenan at concentration of 250 μg/ml. One hour later, the media were removed and total RNA was isolated. (ii) Internalization: MDCK cells were incubated with SW731 at 4°C for 1 h. After removal of the inoculation, the cells were overlaid with infective media in the presence or absence of κ-carrageenan at concentration of 250 μg/ml at 37°C for 1 h, after treatment with protease K for 5 min, total RNA was extracted and analyzed using qRT-PCR. Results were analyzed using the independent sample t test. Values are means ± SEM (n = 3). Significance: *P < 0.05 vs. nondrug treated control group; **P < 0.005 vs. nondrug treated control group; ^*#*^ *P <* 0.05 vs. after adsorption group. Results are representative of two independent experiments.

To further elucidate the mechanism underlying the anti-SW731 activity of κ-carrageenan, we also investigated whether κ-carrageenan elicits its inhibitory actions directly on virus particles, cell receptor, viral adsorption or internalization. MDCK cells were treated with κ-carrageenan at four different conditions, pretreated-SW731, pretreated-cells, during adsorption, and after-adsorption. After 24 h viral titers in the culture media were determined by TCID_50_ assay. Fig 4A shows that pretreated-SW731, SW731 during adsorption, and SW731 after adsorption could all significantly decrease the viral titer in all samples except pretreated cells, suggesting that κ-carrageenan could interfere with binding of SW731 and MDCK by targeting the virus instead of MDCK cells, and blocking internalition. In other cases, both pretreatment-virus and adsorption groups showed significantly lower viral titers than the after-adsorption group, indicating that κ-carrageenan preferentially targets adsorption steps. No difference was observed between the pretreatment-virus and adsorption group, suggesting that κ-carrageenan did not inactivate viral particles. In addition, the number of NP-positive cells in the IFA assay and comparison of mRNA levels confirmed this deduction (Fig 4B and 4C).

To further assess the inhibitory effect of κ-carrageenan on adsorption and internalization, the viral RNA (vRNA) levels were detected in groups treated with or without κ-carrageenan during adsorption and internalization. Fig 4D shows that the vRNA levels of NP in both adsorption and internalization groups treated with κ-carrageenan were significantly lower than in control groups. Taken together, these results suggest that κ-carrageenan had inhibitory actions directly on viral adsorption via target the viral particles instead of cells receptor, and internalization.

### κ-carrageenan specifically inhibited SW731 binding to its sialic acid receptor

**Fig 5:**
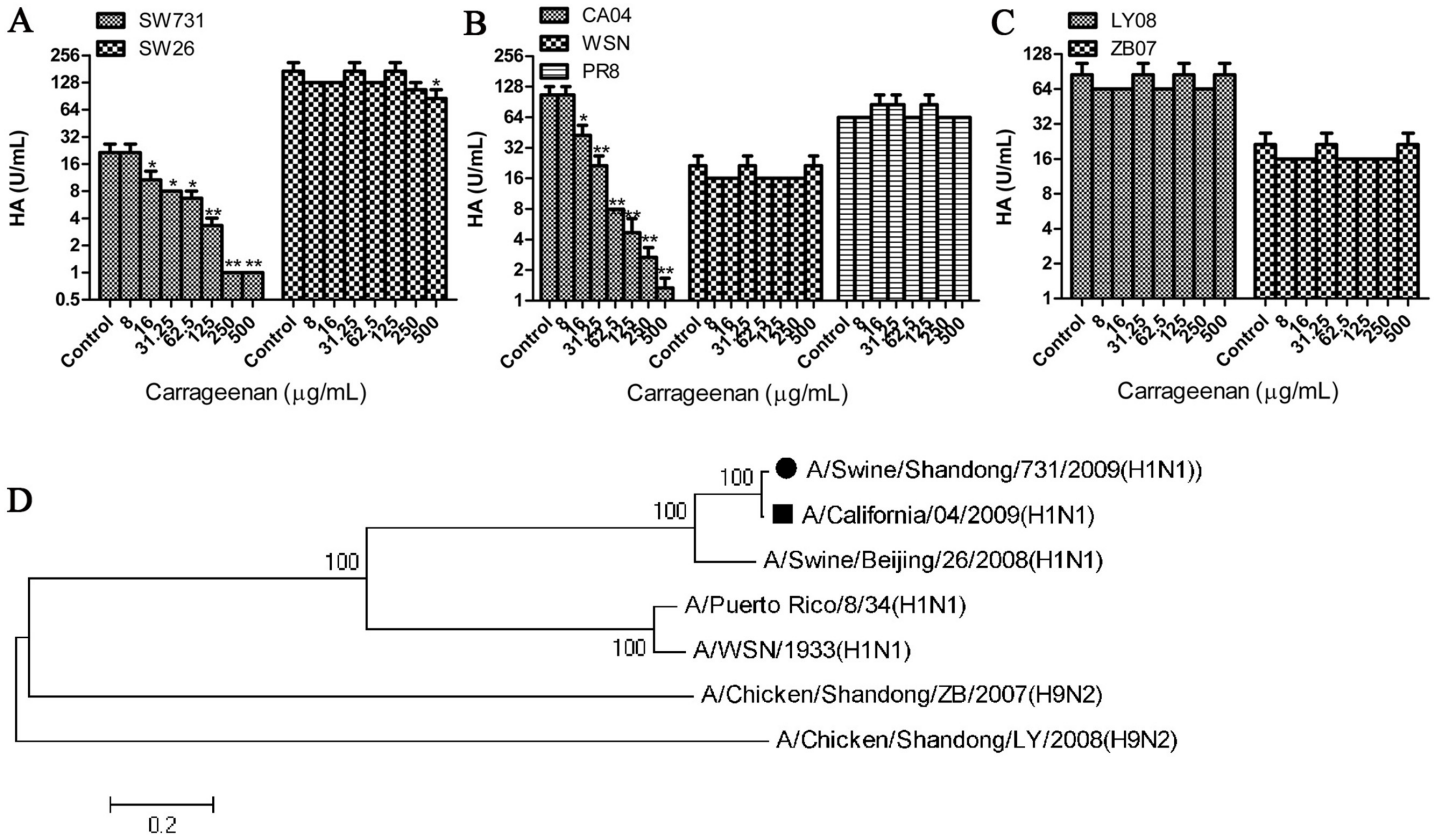
Specific inhibition effect of κ-carrageenan on SW731 HA. (A–C) Twenty-five microliters of serially diluted SW731, SW26, CA04, PR8, WSN, LY08, and ZB07 viruses were incubated with the same volume of PBS (Mock) or serially diluted κ-carrageenan, at RT for 20 min. Then each sample was mixed with 50 μl of 1% chicken red blood cells (RBC). After 30 min, the concentrations of κ-carrageenan inhibiting HA activity were measured. (D) Phylogenetic trees of the HA genes were constructed with sequences from GenBank based on the open reading frame sequences. The tree was generated using the neighbor-joining method in MEGA 6.0, with 1000 bootstrap trials performed to assign confidence to the grouping. Results were analyzed with the independent sample t test (n = 3). Values are means ± SEM. Significance: *P < 0.05 vs. nondrug treated control group. **P < 0.005 vs. nondrug treated control group. Results are representative of two independent experiments.

According to the data shown above, κ-carrageenan interfered with adsorption. It was here determined whether κ-carrageenan could inhibit the binding of HA to its receptors, Neu5Aca2-6Gal-terminated sugar chains. The HA titers of SW731 were markedly reduced by κ-carrageenan in a dose-dependent manner (Fig 5A). To assess the specificity of this inhibitive effect, hemagglutination inhibition assays were also performed on several other influenza viruses. No difference in κ-carrageenan-treated virus PR8, WSN, ZB07, or LY08 was detected in response to PBS treatment, but κ-carrageenan can significantly decrease the HA titer of CA04 (Fig 5B and 5C), which contained a HA sharing 99% amino acid sequence with SW731 (Fig 5D). HA binding of SW26, which has a HA close to SW731 in evolutionary distance, was also slightly blocked by κ-carrageenan at concentration of 500 μg/ml (Fig 5A–5D). These data together indicate that κ-carrageenan specifically inhibited HA of SW731 binding to its sialic acid receptor.

Considering that κ-carrageenan may interact specifically with glycans on the HA protein, we analyzed the potential glycosylation site patterns on HA of 7 strains. As shown in Table 1 and S1 Fig, five potential glycosylation sites at positions 27, 28, 40, 304 and 498 on the stalk were quite conserved in all strains analyzed. There are six potential glycosylation sites in HAs of PR8, ZB07, and LY08, and five in HAs of SW26 and WSN. Only HAs of SW731 and CA04 contain 7 potential glycosylation sites, including two extra carbohydrate attachment sites at 104 and 293 on the head region of HA1, which are not present in other analyzed strains (Table 1). Glycans on glycosylation sites 104 and 293 are close to the receptor binding pocket and the proteolytic activation site of HA, respectively, and might be involved in the inhibitory effect of κ-carrageenan on SW731 binding to its sialic acid receptor.

**Table 1:** Potential glycosylation sites on HA in different influenza viruses.

Location	HA1										HA2	
	Stalk		Side of head			Top of head Side of head		Side of head			Stalk	
	27	28	40	81	104	142	212	286	293	297	304	498
SW731	√	√	√		**√**				**√**		√	√
CA04	√	√	√		**√**				**√**		√	√
SW26	√	√	√				√					√
PR8	√	√	√					√			√	√
WSN	√	√				√		√				√
ZB07		√		√		√				√	√	√
LY08		√		√		√				√	√	√

√ represents the presence of glycosylation sites. The glycosylation sites only present in SW731and CA/04 were highlighted in bold.

To determine whether virus release is modulated by κ-carrageenan, we measured the NA *K*_*m*_ and *V*_max_ values by using the fluorogenic substrate 2′-(4-methylumbelliferyi)-α-d-*N*-acetylneuraminic acid. The *K*_*m*_ values indicated that the κ-carrageenan treatment resulted in a slight decrease in the affinity for the substrate of viruses (Table 2). Similarly, *V*_max_, which was determined by both the specific activity and the amount of enzyme in the reaction mixture, was also little lower for κ-carrageenan treated SW731 than untreated SW731. However, no significant difference was observed in both *K*_*m*_ and *V*_max_ values suggesting that the effect of κ-carrageenan on virus release was not significant.

**Table 2:** Enzyme activity of NA^a^.

	*K*_*m*_ (μM)	*V*_max_
SW731	198.7±22.3	2.49±0.32
SW731+carrageenan	237.9±52.1	2.08±0.24

^a^A standardized virus dose of 10^5^ PFU/ml was used for the NA kinetics assay.

The enzyme kinetics data (± standard deviation) were fit to the Michaelis-Menten equation by nonlinear regression to determine the Michaelis constant (*K*_*m*_) and maximum velocity (*V*_max_) of substrate conversion (in fluorescent units per second).

### κ-carrageenan also inhibited SW731 viral mRNA and protein expression after internalization

**Fig 6:**
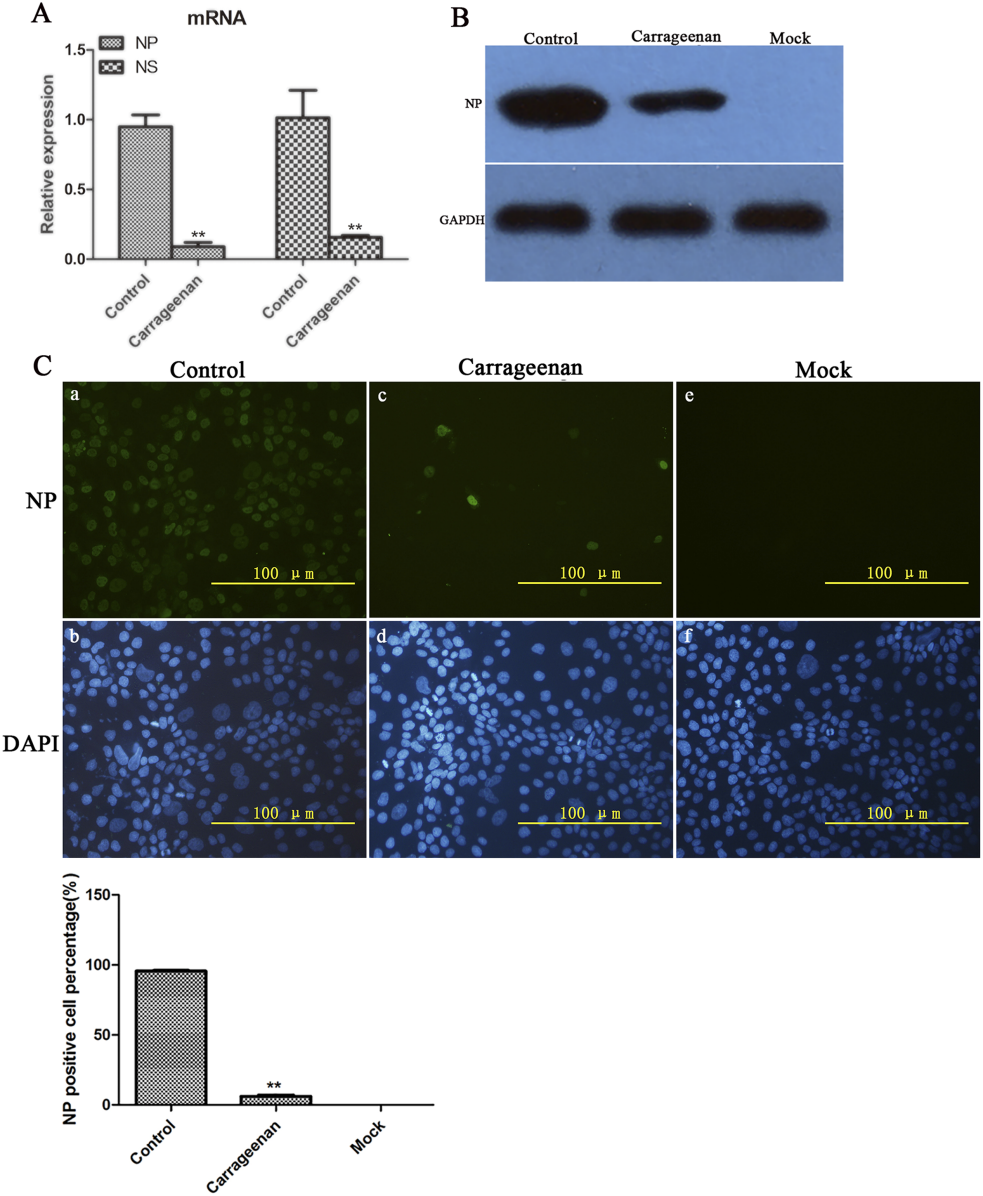
Inhibition effect of κ-carrageenan on SW731 mRNA and protein expression. (A) MDCK cells were infected with SW731 (MOI = 3) for 1h, and then treated with 250 μg/ml of κ-carrageenan or PBS after virus internalization. After 6 h, total RNA was extracted and analyzed using qRT-PCR. (B) MDCK cells were infected with SW731 (MOI = 3), and then treated with 250 μg/ml of κ-carrageenan or PBS after virus internalization. After 8 h, Western blot was performed using anti-NP protein antibody and HRP-labeled second antibody. (C) MDCK cells were infected with SW731 (MOI = 3), and then treated with 250 μg/ml of κ-carrageenan or PBS after viral internalization. After 8 h, immunofluorescence staining was performed using anti-NP protein antibody and FITC-labeled second antibody, and NP positive cell percentage was measured. Results were analyzed with the independent sample t test (n = 3). Values are means ± SEM. Significance: *P < 0.05 vs. nondrug treated control group; **P < 0.005 vs. nondrug treated control group. Results are representative of two independent experiments.

To further study the effect of κ-carrageenan on the replication after internalization, MDCK cells were treated with κ-carrageenan after SW731 infection. After 8 h, total RNA was extracted for qRT-PCR assay. Current data showed that κ-carrageenan treatment reduced SW731 NP mRNA levels to about 9% that of control cells (Fig 6A). SW731 NS1 mRNA levels also decreased to 15% that of untreated control cells after κ-carrageenan treatment (Fig 6A).

The effect of κ-carrageenan on SW731 protein expression was also assessed with Western blot and indirect immunofluorescence assays. MDCK cells were first infected with SW731 (MOI = 3.0) and then treated with κ-carrageenan at a concentration of 250 μg/ml after viral internalization. After 8 h, viral NP protein expression was examined using Western blot and indirect immunofluorescence assays. Fig 6B shows that, in κ-carrageenan-treated MDCK cells, the NP protein level decreased significantly, to 10% that of the control group. No detectable band was found in the mock group. Consistent with the Western blot analysis, indirect immunofluorescence assay showed that almost 95% of the cells in the SW731-infected group not subjected to κ-carrageenan treatment were NP-positive (Fig 6C, a and b), but only 5% cells accumulatin the κ-carrageenan treatment group were (Fig 6C, c and d). No NP-positive cells were detected in mock-treated MDCK cells (Fig 6C, e and f). These data suggested that κ-carrageenan could also inhibit SW731 mRNA and protein expression after viral internalization.

## Discussion

In this report, it was demonstrated that κ-carrageenan, a sulfated polysaccharide extract of red algae, could inhibit replication of SW731 virus by specially interfering with binding of HA to MDCK cells, suppressing mRNA and protein expression after internalization *in vitro*. This indicates that κ-carrageenan may be suitable for use against H1N1/2009 and other viruses containing the HA of H1N1/2009.

Previous reports showed that ι-carrageenan polysaccharides can inhibit replication of HPV [9], influenza A H1N1 virus [15], dengue virus [6], and HRV [8] by blocking the binding of virus to cells. However, Wei Wang et al. demonstrated that κ-carrageenan oligosaccharide suppressed the influenza A H1N1 virus multiplication by directly inactivating viral particles, inhibiting transcription, and protein expression instead of interfering with adsorption [14]. In the current study, adsorption, internalization, transcription, and protein expression of SW731 were all targets of κ-carrageenan, especially the adsorption. Previous reports showed that the inhibitory effect of κ-carrageenan oligosaccharide on WSN was comparable to that observed in PR8 [14]. However, we observed that WSN viral titer was only slightly decreased by κ-carrageenan polysaccharides at a concentration of 250 μg/ml and a low MOI of 0.1. No decrease in PR8, ZB07 viral titer was observed in supernate of MDCK treated with the indicated concentrations of κ-carrageenan polysaccharides. These data suggest that inhibitory effect of κ-carrageenan polysaccharides is strain-specific among H1N1 viruses. Κ-carrageenan polysaccharide and oligosaccharide had different effects on the same influenza virus strain, which might be due to molecular weight and different levels of cell permeability.

Sulfated polysaccharide p-KG03, derived from microalga, *Gyrodinium impudium*, have also been found to inhibit influenza H1N1 virus attachment by interfering with HA and cells [23]. Consistent with this, current data showed that HA of SW731 was inhibited by κ-carrageenan polysaccharide in a dose-dependent manner. The CA04 virus, which contains an HA closely homologous to that of SW731, was also blocked in HA assay. However, κ-carrageenan polysaccharide only slightly decreased the HA titer of SW26 at concentration of 500 μg/ml. PR8, WSN, LY08, and ZB07 HA titer were not influenced by κ-carrageenan polysaccharide. In addition, the pretreated cells could not decrease the SW731 viral titer. These results indicate that κ-carrageenan polysaccharide might specifically target the SW731 HA instead of blocking the receptor on cells. The groups pretreated with SW731 and adsorption showed comparable viral titers and mRNA and protein levels, which suggest that κ-carrageenan may interact solely with HA of SW731 without inactivating viral particles.

Glycans near the proteolytic activation site of HA modulate HA0 cleavage and influence the infectivity of influenza virus [24]. Viral receptor binding also requires HA glycosylation [25]. Our analysis of HA sequences revealed that SW731 and CA04 contain two extra carbohydrate attachment site at 104 on the head region of HA1, and 293 close to the proteolytic activation site of HA compared with the rest of strains. However, whether κ-carrageenan interfere with adsorption and internalization via binding with the glycans at positions 104 or 293 still need further research.

Our Figs 3 and 4 demonstrated that internalization of SW731 was blocked by κ-carrageenan. However, whether the membrane fusion is influenced by κ-carrageenan is not clear. Given the optimal pH of fusion for the different viruses are not the same. The influence of κ-carrageenan on pH optima of SW731 is also a potential cause for replication inhibitory effect.

In addition to blocking the adsorption and internalization, κ-carrageenan also inhibits transcription and protein expression. Low-molecular-weight carrageenan can enter MDCK cells and target several of the intracellular steps of the viral life cycle [14]. However, high-molecular-weight carrageenan has poor permeability and instead targets the attachment rather than intracellular steps [26]. The κ-carrageenan used here included both low- and high-molecular-weight κ-carrageenan. The inhibitory effect on mRNA transcription and protein expression could be due to the interference of polymerase activity by low-molecular-weight κ-carrageenan in cells. However, the high-molecular-weight κ-carrageenan can interact directly with the HA of SW731 to rob the virus of its ability to infect cells.

The intracellular events, caused by κ-carrageenan, indicate that κ-carrageenan might influence uncoating, nucleus import, nucleus export, or host antiviral innate immune response. Bae et al. and Yim et al. reported that sulfated polysaccharides can activate nitric oxide production in a JNK-dependent manner and stimulate the production of cytokines, such as interleukin-1 (IL-1), IL-6, and TNF-α in macrophages [27,28]. Zhou Ge-fei et al. demonstrated that κ-carrageenan could also increase NO production in murine peritioneal macrophages [29]. As shown in S2 Fig, the κ-carrageenan could decrease the *IL-6* and *TNF-α* mRNA levels but promote the *IL-1β* mRNA synthesis with or without SW731 infection. These data indicate that κ-carrageenan may also regulate the signaling pathways involved in cytokine production. However, the effect of κ-carrageenan on cytokine synthesis was not observed in A549 cells (data not shown), which imply that CPE inhibitory effect of κ-carrageenan is not via blocking host cytokines.

Considering that the higher concentration (EC_50_ value = 89.57±2.1 μg/ml) for effectiveness compared with Ribavirin (EC_50_ value = 8.3 μg/ml) may limit its application, further separation and purification of certain molecular-weight κ-carrageenan for anti-H1N1/2009 viruses research will be needed.

In summary, these results suggest that κ-carrageenan is a safe and effective means of inhibiting SW731 virus infection *in vitro*. Research into the underlying mechanism showed that κ-carrageenan inhibited replication mainly by specifically blocking the binding of HA to MDCK cells, internalization, mRNA and protein expression instead of blocking host cell receptor and viral release. κ-carrageenan is a promising antiviral candidate for the treatment of influenza H1N1/2009 viruses and other strains containing the homologous HA.

## Supporting Information

S1 Fig Analysis of potential glycosylation sites on HA of seven influenza viruses. The NXT/S motif in amino acid sequences of seven HA proteins were analyzed using DNAMAN. All the NXT/S motifs have been marked with red frames.
(BMP)

S2 Fig Cytokine regulation by κ-carrageenan in RAW 264.7 cells. (A–C) RAW cells were first either infected with SW731 virus or mock-treated and then treated with κ-carrageenan or PBS after adsorption. After 6 h, total RNA was extracted and analyzed with qRT-PCR. Results were analyzed with the independent sample t test (n = 3). Values are means ± SEM. Significance: *P < 0.05 vs. nondrug treated control group; **P < 0.005 vs. nondrug treated control group. Results are representative of two independent experiments.
(EPS)

S1 Table Primers list. (DOCX)
