# Supplementary figures and images for: Specific Inhibitory Effect of κ-Carrageenan Polysaccharide on Swine Pandemic 2009 H1N1 Influenza Virus
Source: PLoS One. 2015 May 13;10(5):e0126577. doi: 10.1371/journal.pone.0126577 (PMC4430168; doi:10.1371/journal.pone.0126577)

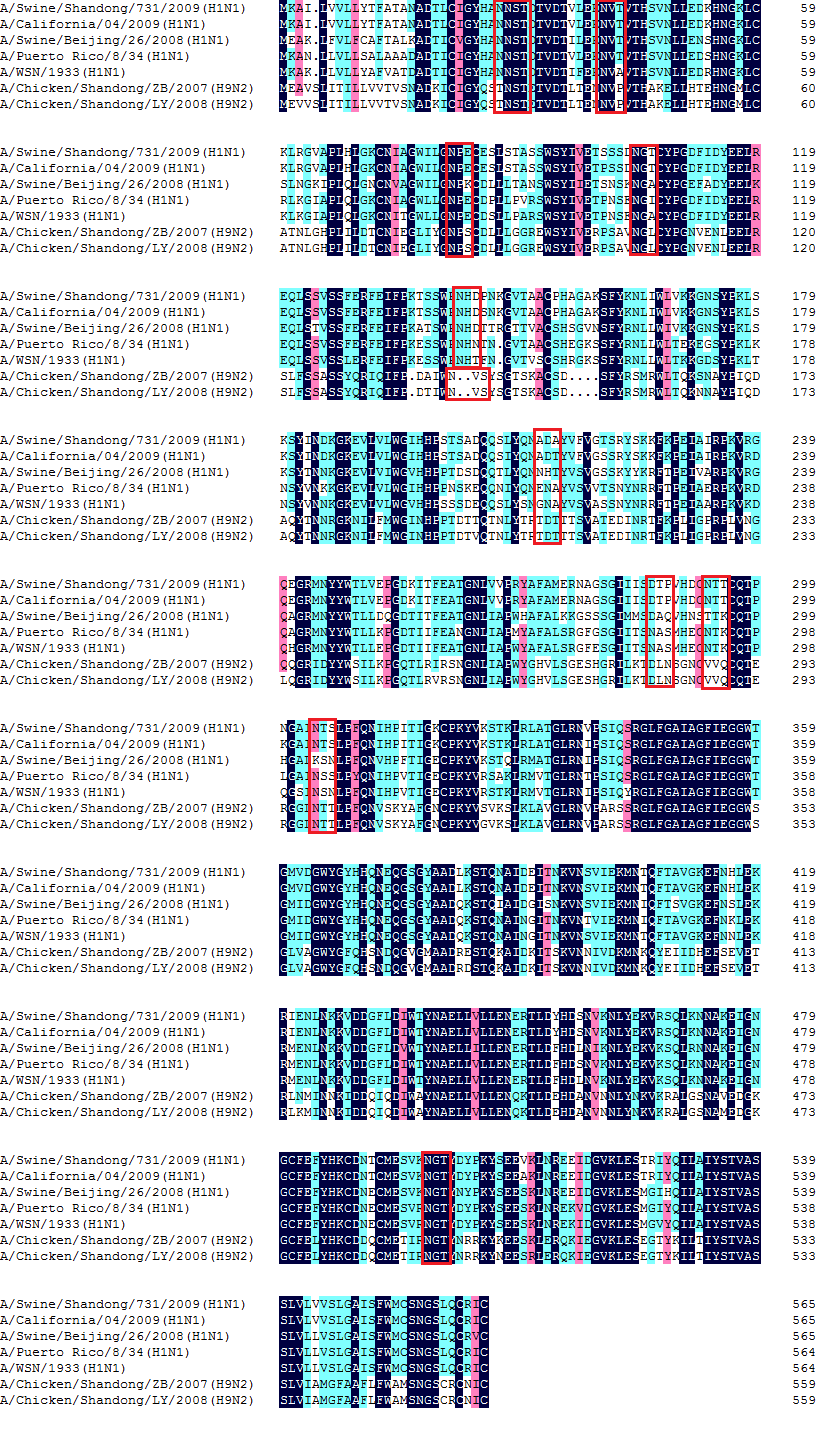

Supplement: S1 Fig — The NXT/S motif in amino acid sequences of seven HA proteins were analyzed using DNAMAN. All the NXT/S motifs have been marked with red frames. (BMP) [file pone.0126577.s001.bmp]
