# Supplementary material for: Specific Inhibitory Effect of κ-Carrageenan Polysaccharide on Swine Pandemic 2009 H1N1 Influenza Virus
Source: PLoS One. 2015 May 13;10(5):e0126577. doi: 10.1371/journal.pone.0126577 (PMC4430168; doi:10.1371/journal.pone.0126577)
Supplement: S1 Table — (DOCX) [file pone.0126577.s003.docx]

**Table S1. Primers list**

| Genes | Direction | Sequence | AccessionNO.In GenBank |
| --- | --- | --- | --- |
| GAPDH | Forward | CCGTGTTCCTACCCCCAAT | AK168217.1 |
|  | Reverse | TGTCATCATACTTGGCAGGTTTCT |  |
| IL-1β | Forward | GAAATGCCACCTTTTGACAGTG | NM_008361.3 |
|  | Reverse | TGGATGCTCTCATCAGGACAG |  |
| IL-6 | Forward | TCGGAGGCTTAATTACACATGTTCT | NM_031168.1 |
|  | Reverse | TGCCATTGCACAACTCTTTTCT |  |
| TNF-α | Forward | CCCCAAAGGGATGAGAAGTTC | NM_013693.2 |
|  | Reverse | GTGTGAGGGTCTGGGCCATA |  |
